# The prognostic nutritional index (PNI) and sepsis-induced cardiomyopathy (SCM) risk in the ICU: a retrospective study with L-shaped analysis

**DOI:** 10.3389/fnut.2026.1766961

**Published:** 2026-05-04

**Authors:** Shuai Wang, Guannan Liu, Jianguo Zhang

**Affiliations:** 1 Department of Emergency, Linyi People's Hospital, Linyi, Shandong, China; 2 Department of Critical Care Medicine, Linyi People's Hospital, Linyi, Shandong, China

**Keywords:** ICU, PNI, prognostic nutritional index, sepsis, sepsis-induced cardiomyopathy

## Abstract

**Background:**

Sepsis-induced cardiomyopathy (SCM) is a common complication in patients with sepsis. Although the prognostic nutritional index (PNI) is less comprehensive than other assessment methods, it remains a readily accessible indicator of nutritional and immune status. This study aimed to investigate the relationship between SCM and the PNI.

**Methods:**

This retrospective study included 200 septic patients admitted to the intensive care unit (ICU) at Linyi City People’s Hospital from 2023 to 2025. The primary endpoint was the occurrence of SCM during the ICU stay. Patients lacking essential variables (e.g., N-terminal pro-B-type natriuretic peptide [NT-proBNP] and ejection fraction [EF]) were excluded from the study. Missing data for other variables were imputed using multiple imputation methods. Restricted cubic spline (RCS) analysis and multivariate logistic regression analyses were performed to evaluate the relationship between SCM and the PNI. Subgroup analyses validated the robustness of the findings.

**Results:**

SCM occurred in 63% of the study population, with incidence rates of 83.33, 61.19, and 44.78% in the T1 (PNI < 31.3), T2 (31.3 ≤ PNI < 38.65), and T3 (PNI ≥ 38.65) groups, respectively. A multivariate logistic regression analysis revealed a significant inverse association between the PNI and SCM (OR = 0.909, 95% CI: 0.862–0.955). Compared with the T1 group, the T3 and T2 groups had significantly lower SCM risks (OR = 0.162, 95% CI: 0.069–0.356; OR = 0.315, 95% CI: 0.135–0.700, respectively). The RCS analysis demonstrated an L-shaped relationship between the PNI and SCM risk, indicating a 10% reduction in risk per unit increase in PNI when the PNI was below 35. Subgroup analyses confirmed these results.

**Conclusion:**

The PNI showed a negative, L-shaped association with the risk of SCM. These findings suggest that PNI can serve as an early risk stratification tool for SCM in septic patients, facilitating timely clinical intervention.

## Introduction

Infection triggers a dysregulated host response, resulting in sepsis, a life-threatening condition characterized by organ dysfunction that significantly affects patient survival and quality of life globally (1). Data from high-income countries initially estimated approximately 31.5 million sepsis cases and 19.4 million cases of severe sepsis worldwide annually, potentially causing 5.3 million deaths per year (2). Recent epidemiological updates emphasize that sepsis represents a substantial proportion (10–25%) of all ICU admissions globally (3, 4), placing immense economic burdens on healthcare systems (3). Despite increased research into sepsis, difficulties persist in its identification and management (5).

Sepsis-induced cardiomyopathy (SCM), a common complication characterized by myocardial dysfunction resulting from sepsis, has attracted sustained attention from cardiologists and intensivists (6). Recent studies have indicated that SCM occurs in 10–60% of septic patients admitted to ICUs, significantly increasing the risk of adverse clinical outcomes (6). Although SCM lacks a standardized definition, there is consensus regarding its reversibility (7). However, several studies have reported that SCM occurrence is associated with a two- to three-fold increase in mortality among septic patients (8, 9). Predicting SCM onset remains challenging, despite established associations between SCM prognosis and scoring systems. These systems include the Sequential Organ Failure Assessment (SOFA), which assesses organ dysfunction severity, and the Acute Physiology and Chronic Health Evaluation II (APACHE II), which classifies disease severity and predicts mortality (1). Therefore, identifying indicators associated with SCM development in septic patients is critical for facilitating timely therapeutic interventions.

Immunodeficiency and malnutrition frequently coexist in septic patients, significantly affecting their prognosis (10, 11). Although the PNI was initially developed to assess preoperative nutritional status and predict postoperative complications (12), its application has expanded considerably to general ICU populations (13). Systematic nutritional screening and assessment in ICUs are essential, as malnutrition significantly influences clinical outcomes in critically ill patients, regardless of surgical status (13). Recent epidemiological data have shown that malnutrition affects 38–78% of ICU patients upon admission (13). Calculated from serum albumin and lymphocyte count, the PNI serves as a practical bedside indicator integrating nutritional and immune status in high-risk populations (14–16). Recent studies have demonstrated associations between the PNI and outcomes in chronic kidney disease and cardiovascular disease (17, 18). Additionally, reduced PNI is associated with poor outcomes in cancer patients (12). While the prognostic value of the PNI in various sepsis-related contexts has been documented, particularly regarding overall mortality and clinical deterioration (11, 19), research into its relationship with SCM remains limited. Existing evidence has indicated that low PNI potentially predicts poor outcomes in septic patients, reflecting nutritional-immune exhaustion and predisposing individuals to multi-organ dysfunction (19, 20).

Therefore, this study aimed to investigate the association between SCM and the PNI, providing methods and clinical guidance for early interventions targeting nutrition and immunity in SCM patients. These measures could improve outcomes and reduce mortality among septic patients.

## Methods

### Data sources

This retrospective study included the clinical data of patients hospitalized in the ICU at Linyi City People’s Hospital between 2023 and 2025. The collected data comprised vital signs, diagnostic details, medication records, demographic characteristics, and laboratory test results. Ethical approval was granted by the Ethics Committee of Linyi City People’s Hospital. The study strictly complied with the ethical principles outlined in the Declaration of Helsinki. Routine clinical procedures were performed throughout the study, and all identifying patient information was removed. Due to the retrospective nature of this study, informed consent was not required.

### Study design and population

**Figure 1:**
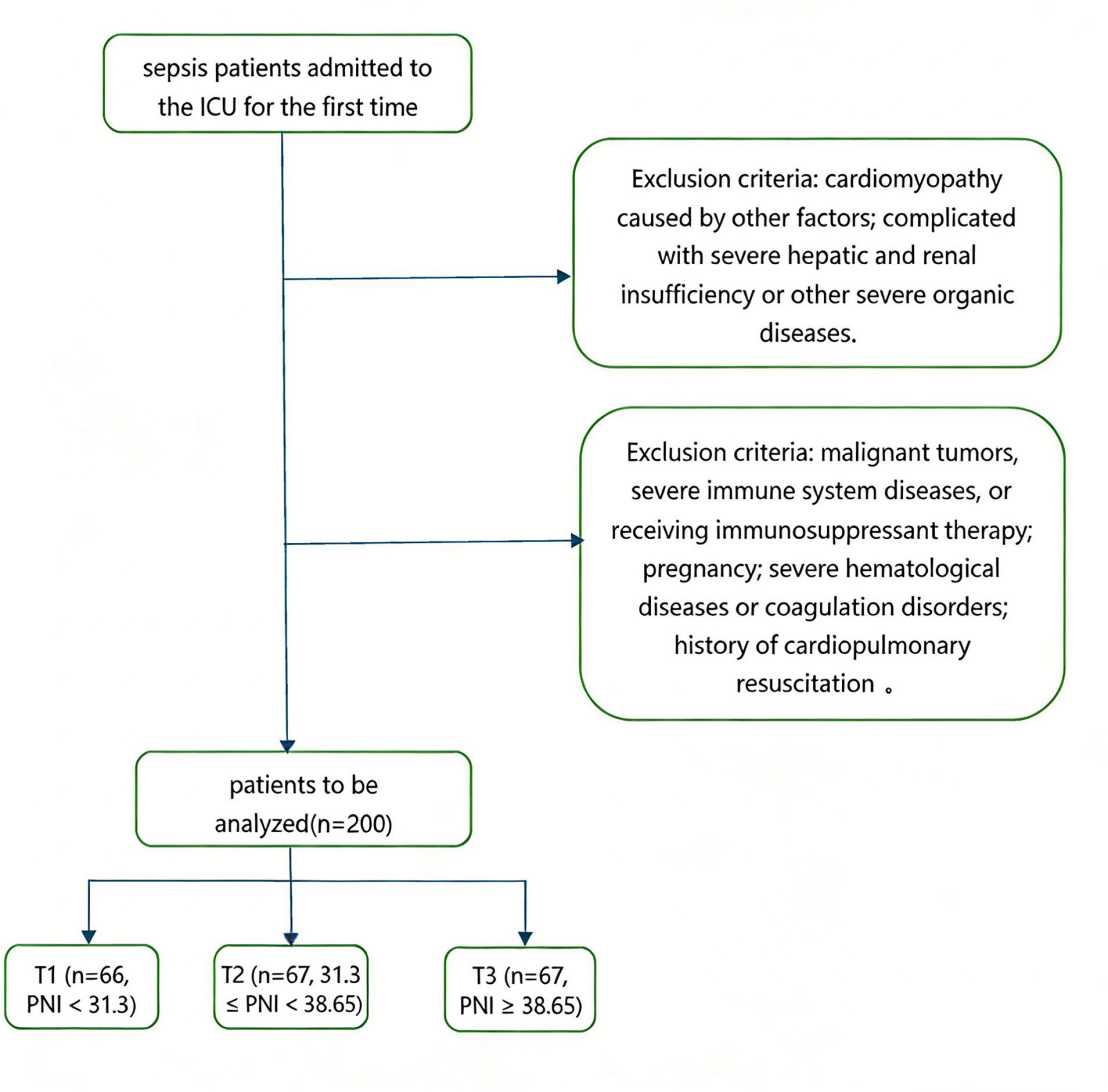
Flowchart of subject selection.

From the hospital’s medical record system, 200 patients with sepsis who were admitted to the ICU for the first time were included. The inclusion criteria were as follows: (1) patients admitted to the ICU with sepsis for the first time; (2) patients aged ≥18 years; and (3) patients whose ICU stay exceeded 70 h. The exclusion criteria were as follows: (1) patients diagnosed with cardiomyopathy due to other causes, such as myocarditis or ischemic heart disease; patients with severe renal insufficiency (estimated glomerular filtration rate [eGFR] < 15 mL/min/1.73 m^2^ or dialysis-dependent) or severe hepatic insufficiency (Child-Pugh Class C or decompensated cirrhosis); (2) patients with malignant tumors, severe immune disorders, or those undergoing immunosuppressive therapy; (3) patients who were pregnant; (4) patients exhibiting coagulation abnormalities or severe hematologic disorders; and (5) patients who underwent cardiopulmonary resuscitation postoperatively. Ultimately, 200 eligible participants were enrolled and divided into three groups according to PNI tertiles (Figure 1).

### Sample size selection

Sample size estimation was based on a previously reported SCM incidence of approximately 60% in septic patients. Assuming an odds ratio (OR) of 0.91 for the association between the PNI and SCM, with *α* = 0.05 (one-sided) and a power (1 − β) of 0.80, adjustments were made for a 10% dropout rate and 15 covariates. This yielded an initial estimate of 150 cases (10 events per covariate), which was revised to 165 cases after dropout adjustment. Therefore, a minimum of 170 cases was required, and 200 eligible patients were ultimately selected for statistical analysis.

### Data collection

Data extracted from the hospital’s medical record system included (1) demographic information: body mass index (BMI), sex, and age at admission; (2) medical history: chronic obstructive pulmonary disease, chronic kidney disease, diabetes mellitus, and hypertension; (3) vital signs: mean blood pressure, respiratory rate, heart rate, and temperature; (4) laboratory parameters: D-dimer, platelet count, white blood cell count, albumin, lymphocytes, and pH; (5) in-hospital medications: vasopressors; (6) procedures performed: mechanical ventilation, emergency surgery, continuous renal replacement therapy (CRRT), and blood transfusion; (7) disease severity scores: SOFA and APACHE II; and (8) hospitalization duration and outcomes: ICU days and hospital days. Blood biochemical samples were collected before therapeutic interventions and within 24 h of ICU admission. Multiple imputation using the random forest approach (via the mice package in R software, with five standard iterations) was performed for variables with less than 20% missing data. Data management and cleaning were conducted using the tidyr and dplyr packages. To prevent bias, variables with more than 20% missing data were excluded. Imputed datasets were used for RCS analysis and multivariate logistic regression analysis to ensure reliability.

### Outcome variables

Patients admitted to the ICU with sepsis were included in this study, and the diagnosis of sepsis was confirmed using the Sepsis-3.0 criteria (1). The criteria for diagnosing SCM included NT-proBNP levels >450 ng/L in patients aged <50 years, >900 ng/L in patients aged 50–75 years, and >1,800 ng/L in patients aged >75 years; and/or a left ventricular ejection fraction (LVEF) of <50% within 24 h after the sepsis diagnosis (21).

### PNI calculation

The PNI was calculated as [10 × serum albumin (g/dL) + 0.005 × peripheral blood lymphocyte count (10^9^/L)] using the initial measurement obtained within 24 h of ICU admission in septic patients.

### Statistical analysis

Normality tests were initially performed on all continuous variables. Except for heart rate, all variables showed non-normal distributions. Variables with normally distributed data were represented as the mean ± standard deviation. Non-normally distributed data were expressed as the median and interquartile range (IQR). The Kruskal–Wallis rank-sum test was applied for intergroup comparisons. Categorical variables were described using frequencies and percentages, and Pearson’s chi-squared test was used for comparisons between the groups.

Analysis of interleukin-6 (IL-6) data revealed an excessively broad IQR, which could affect the accuracy of subsequent statistical analyses. To address this, the IL-6 values underwent log_10_ transformation (log_10_(IL-6)) using R software before further analysis. Transformed IL-6 data that remained non-normally distributed were reported as median and IQR.

Participants were divided into three groups based on PNI tertiles: T1 (<31.3, *n* = 66), T2 (31.3–38.65, *n* = 67), and T3 (≥38.65, *n* = 67). Multicollinearity tests were performed on all variables included in the regression models, and variables with variance inflation factors above 5 were excluded. A multivariate logistic regression analysis was used to investigate the association between the PNI and newly developed SCM in patients with sepsis. The non-adjusted model excluded any additional variables. Adjusted Model I included BMI, sex, and age. Adjusted Model II further included clinically relevant variables such as blood transfusion, CRRT, vasopressor use, chronic obstructive pulmonary disease, chronic kidney disease, hypertension, diabetes mellitus, log10(IL-6), white blood cell count, SOFA, and APACHE II scores.

An RCS analysis was used to examine potential non-linear associations between the PNI and SCM incidence. Subgroup analyses were performed based on vasopressor use, chronic obstructive pulmonary disease, chronic kidney disease, hypertension, diabetes mellitus, age (<65 years or ≥65 years), and sex (male patients/female patients).

All statistical analyses were conducted using R software (version 4.3.2, R Foundation for Statistical Computing, Austria), and statistical significance was defined as a *p*-value of < 0.05.

## Results

This study included 200 patients with sepsis (36% female patients, 64% male patients), with a median age of 69 years and an overall SCM incidence of 63%. The frequency of SCM was 83.33% in T1, 61.19% in T2, and 44.78% in T3. Participants were categorized into three groups according to PNI tertiles: T1 (<31.3, *n* = 66), T2 (31.3–38.65, *n* = 67), and T3 (≥38.65, *n* = 67). Table 1 shows the baseline characteristics, including indirect bilirubin (IBIL), chronic obstructive pulmonary disease (COPD), C-reactive protein (CRP), platelets (PLT), and blood urea nitrogen (BUN), which exhibited significant differences between the groups. The frequency of COPD was 12.12% in T1 and 1.49% in T3 (*p* = 0.032). Patients with higher PNI (T3) exhibited lower BUN levels, lower COPD prevalence, and lower CRP levels, in addition to higher bilirubin and platelet counts.

**Table 1:** Baseline parameters comparisons of patients with sepsis and SCM.

Characteristic	Overall	T1	T2	T3	*p*-value
	*N* = 200	PNI < 31.3, *N* = 66	31.3 ≤ PNI < 38.65, *N* = 67	PNI ≥ 38.65, *N* = 67	
Sex, *n* (%)					0.197
Male	72 (36.00%)	24 (36.36%)	29 (43.28%)	19 (28.36%)	
Female	128 (64.00%)	42 (63.64%)	38 (56.72%)	48 (71.64%)	
Age (year)	69.00 (55.00, 77.50)	70.50 (62.00, 78.00)	67.00 (52.00, 77.00)	65.00 (54.00, 79.00)	0.107
Temperature (°C)	36.60 (36.50, 37.00)	36.60 (36.50, 37.00)	36.50 (36.50, 36.80)	36.60 (36.50, 37.20)	0.249
Heart rate (bpm)	96.81 ± 19.36	96.26 ± 18.71	96.31 ± 18.99	97.84 ± 20.57	0.967
Respiratory rate (bpm)	20.00 (17.00, 24.00)	20.00 (18.00, 25.00)	20.00 (16.00, 25.00)	20.00 (17.00, 24.00)	0.792
MAP (mmHg)	89.00 (76.00, 103.50)	89.00 (76.00, 100.00)	87.00 (72.00, 98.00)	93.00 (77.00, 109.00)	0.250
BMI (kg/m^2^)	23.80 (21.20, 27.05)	23.00 (20.30, 26.70)	23.50 (21.60, 26.10)	24.20 (21.50, 27.70)	0.373
APACHE II	16.00 (14.00, 20.00)	16.00 (13.00, 22.00)	16.00 (14.00, 20.00)	16.00 (15.00, 19.00)	0.868
SOFA	5.00 (3.00, 8.00)	6.50 (4.00, 9.00)	5.00 (3.00, 8.00)	5.00 (3.00, 7.00)	0.071
LAC (mmol/L)	1.70 (1.10, 2.90)	1.80 (1.10, 2.80)	1.80 (1.20, 3.40)	1.40 (1.10, 2.10)	0.143
OI (mmHg)	276.00 (200.00, 359.50)	248.00 (200.00, 342.00)	276.00 (193.00, 358.00)	300.00 (210.00, 379.00)	0.229
WBC (×10^9^/L)	12.56 (7.31, 17.65)	12.40 (6.42, 16.50)	12.71 (7.12, 17.33)	14.21 (9.94, 21.10)	0.164
PLT (×10^9^/L)	172.00 (90.50, 263.00)	154.50 (54.00, 250.00)	156.00 (94.00, 236.00)	205.00 (120.00, 295.00)	0.018
D-dimer (ng/mL)	105.15 (49.40, 192.30)	126.65 (73.50, 194.00)	110.80 (51.20, 184.00)	87.00 (34.50, 198.10)	0.181
CRP (mg/L)	4.02 (2.08, 8.54)	4.58 (2.80, 8.33)	4.29 (2.21, 10.02)	2.81 (1.07, 6.71)	0.015
TNT (ng/mL)	0.04 (0.01, 0.10)	0.04 (0.02, 0.11)	0.03 (0.01, 0.09)	0.03 (0.01, 0.08)	0.250
CK-MB (ng/mL)	2.59 (1.27, 5.46)	2.28 (1.19, 4.97)	3.16 (1.10, 7.58)	2.63 (1.46, 4.92)	0.508
PCT (ng/mL)	7.76 (1.32, 45.67)	17.88 (1.55, 60.10)	11.48 (1.94, 51.62)	5.08 (0.79, 40.22)	0.130
BUN (mmol/L)	12.90 (7.55, 21.20)	16.70 (9.60, 23.10)	11.90 (8.10, 21.10)	11.40 (5.90, 16.70)	0.022
CR (μmol/L)	132.00 (75.00, 270.00)	156.00 (73.00, 282.00)	134.00 (77.00, 340.00)	115.00 (81.00, 199.00)	0.534
TBIL (μmol/L)	13.85 (7.95, 23.70)	12.60 (6.80, 26.00)	14.00 (7.70, 20.20)	14.70 (8.10, 24.00)	0.580
DBIL (μmol/L)	8.15 (4.55, 14.35)	9.00 (4.90, 17.60)	7.40 (4.20, 13.20)	8.10 (4.70, 13.50)	0.445
IBIL (μmol/L)	4.40 (2.65, 8.00)	3.30 (2.10, 6.10)	4.20 (2.40, 8.00)	5.60 (3.60, 9.10)	0.003
AST (U/L)	30.60 (18.95, 57.60)	30.60 (19.90, 47.50)	32.60 (18.50, 57.60)	28.70 (16.90, 67.70)	0.961
ALT (U/L)	23.15 (13.75, 48.25)	23.45 (13.40, 40.50)	22.00 (15.00, 57.80)	23.20 (13.80, 60.00)	0.873
IL-6 (pg/mL)	252.20 (56.60, 2,500.00)	395.66 (63.63, 1,475.17)	139.18 (37.79, 2,500.00)	295.52 (88.21, 2,500.00)	0.495
Log_10_ (IL-6)	5.53 (4.04, 7.82)	5.98 (4.15, 7.30)	4.94 (3.63, 7.82)	5.69 (4.48, 7.82)	0.491
EF (%)	58.00 (55.00, 58.00)	58.00 (54.00, 58.00)	58.00 (55.00, 58.00)	58.00 (55.00, 58.00)	0.318
PNI	35.28 (31.13, 38.65)	29.35 (27.45, 31.10)	35.25 (33.75, 36.40)	41.05 (38.65, 44.10)	<0.001
Diabetes, *n* (%)					0.071
No	142 (71.00%)	46 (69.70%)	42 (62.69%)	54 (80.60%)	
Yes	58 (29.00%)	20 (30.30%)	25 (37.31%)	13 (19.40%)	
Hypertension, *n* (%)					0.506
No	120 (60.00%)	38 (57.58%)	38 (56.72%)	44 (65.67%)	
Yes	80 (40.00%)	28 (42.42%)	29 (43.28%)	23 (34.33%)	
CKD, *n* (%)					0.939
No	184 (92.00%)	61 (92.42%)	61 (91.04%)	62 (92.54%)	
Yes	16 (8.00%)	5 (7.58%)	6 (8.96%)	5 (7.46%)	
COPD, *n* (%)					0.032
No	188 (94.00%)	58 (87.88%)	64 (95.52%)	66 (98.51%)	
Yes	12 (6.00%)	8 (12.12%)	3 (4.48%)	1 (1.49%)	
Shock, *n* (%)					0.377
No	69 (34.50%)	19 (28.79%)	27 (40.30%)	23 (34.33%)	
Yes	131 (65.50%)	47 (71.21%)	40 (59.70%)	44 (65.67%)	
Pressor agent, *n* (%)					0.800
No	76 (38.00%)	23 (34.85%)	26 (38.81%)	27 (40.30%)	
Yes	124 (62.00%)	43 (65.15%)	41 (61.19%)	40 (59.70%)	
ARDS, *n* (%)					0.706
No	156 (78.00%)	53 (80.30%)	50 (74.63%)	53 (79.10%)	
Yes	44 (22.00%)	13 (19.70%)	17 (25.37%)	14 (20.90%)	
AKI, *n* (%)					0.074
No	98 (49.00%)	31 (46.97%)	27 (40.30%)	40 (59.70%)	
Yes	102 (51.00%)	35 (53.03%)	40 (59.70%)	27 (40.30%)	
AF, *n* (%)					0.715
No	178 (89.00%)	60 (90.91%)	58 (86.57%)	60 (89.55%)	
Yes	22 (11.00%)	6 (9.09%)	9 (13.43%)	7 (10.45%)	
MV, *n* (%)					0.360
No	75 (37.50%)	25 (37.88%)	21 (31.34%)	29 (43.28%)	
Yes	125 (62.50%)	41 (62.12%)	46 (68.66%)	38 (56.72%)	
Emergency surgery, *n* (%)					0.229
No	152 (76.00%)	55 (83.33%)	48 (71.64%)	49 (73.13%)	
Yes	48 (24.00%)	11 (16.67%)	19 (28.36%)	18 (26.87%)	
Transfusion, *n* (%)					0.912
No	110 (55.00%)	35 (53.03%)	37 (55.22%)	38 (56.72%)	
Yes	90 (45.00%)	31 (46.97%)	30 (44.78%)	29 (43.28%)	
CRRT, *n* (%)					0.736
No	161 (80.50%)	52 (78.79%)	53 (79.10%)	56 (83.58%)	
Yes	39 (19.50%)	14 (21.21%)	14 (20.90%)	11 (16.42%)	
Outcome, *n* (%)					0.761
Death	9 (4.50%)	5 (7.58%)	2 (2.99%)	2 (2.99%)	
Withdrawal of active treatment	55 (27.50%)	18 (27.27%)	19 (28.36%)	18 (26.87%)	
Clinical recovery and discharge alive	136 (68.00%)	43 (65.15%)	46 (68.66%)	47 (70.15%)	
Infection sites, *n* (%)					0.296
Pulmonary	72 (36.00%)	30 (45.45%)	24 (35.82%)	18 (26.87%)	
Abdominal	91 (45.50%)	26 (39.39%)	33 (49.25%)	32 (47.76%)	
Genitourinary	18 (9.00%)	6 (9.09%)	4 (5.97%)	8 (11.94%)	
Other sites	19 (9.50%)	4 (6.06%)	6 (8.96%)	9 (13.43%)	
SCM, *n* (%)					<0.001
No	74 (37.00%)	11 (16.67%)	26 (38.81%)	37 (55.22%)	
Yes	126 (63.00%)	55 (83.33%)	41 (61.19%)	30 (44.78%)	
In-hospital stay duration (days)	12.00 (8.00, 18.00)	11.00 (8.00, 17.00)	12.00 (9.00, 19.00)	12.00 (8.00, 18.00)	0.660
ICU stay duration (days)	6.00 (3.50, 12.00)	5.00 (4.00, 12.00)	8.00 (4.00, 12.00)	6.00 (3.00, 12.00)	0.453

BMI, body mass index; APACHE II, acute physiology and chronic health evaluation II; SOFA, sequential organ failure assessment; LAC, lactic acid; OI, oxygenation index; WBC, white blood cell count; PLT, platelet count; CRP, C-reactive protein; TNT, troponin T; CK-MB, creatine kinase-MB; PCT, procalcitonin; BUN, blood urea nitrogen; CR, creatinine; TBIL, total bilirubin; DBIL, direct bilirubin; IBIL, indirect bilirubin; AST, aspartate aminotransferase; ALT, alanine aminotransferase; IL-6, interleukin-6; EF, ejection fraction; PNI, prognostic nutritional index; CKD, chronic kidney disease; COPD, chronic obstructive pulmonary disease; ARDS, acute respiratory distress syndrome; AKI, acute kidney injury; AF, atrial fibrillation; MV, mechanical ventilation; CRRT, continuous renal replacement therapy; SCM, sepsis-induced cardiomyopathy.

### Study results

Three multivariate logistic regression models were used in this study to examine the association between the PNI and SCM development. The unadjusted model did not include any confounding factors. Adjusted Model I included adjustments for demographic factors such as BMI, sex, and age. Adjusted Model II further incorporated clinically relevant factors, including blood transfusion, CRRT, vasopressor use, chronic obstructive pulmonary disease, chronic kidney disease, hypertension, diabetes mellitus, IL-6 levels, white blood cell count, SOFA score, and APACHE II score.

In the unadjusted model, each one-unit increase in PNI was associated with a significant 10% reduction in SCM risk (OR = 0.909, 95% CI: 0.862–0.955, *p* < 0.001). This protective effect remained significant after adjusting for confounders. In Adjusted Model I, the OR was 0.912 (95% CI: 0.863–0.960, p < 0.001). In Adjusted Model II (fully adjusted), the OR was 0.904 (95% CI: 0.848–0.961, *p* = 0.002). PNI grouping was also significantly associated with outcomes in all models. The results suggest that the PNI is an independent protective factor against SCM in septic patients, with a graded decrease in SCM risk as PNI levels increase (T3 > T2 > T1). This association remained statistically significant after controlling for multiple confounders, indicating that nutritional status may influence SCM development through various pathways (Table 2).

**Table 2:** Logistic regression analysis of the association between the PNI and SCM occurrence in patients with sepsis.

Variable	Non-adjusted		AdjustI		AdjustII	
	OR (95% CI)	*p*-value	OR (95% CI)	*p*-value	OR (95% CI)	*p*-value
PNI	0.909 (0.862, 0.955)	<0.001	0.912 (0.863, 0.960)	<0.001	0.904 (0.848, 0.961)	0.002
T1	—		—		—	
T2	0.315 (0.135, 0.700)	0.006	0.315 (0.132, 0.712)	0.006	0.304 (0.110, 0.789)	0.018
T3	0.162 (0.069, 0.356)	<0.001	0.169 (0.071, 0.378)	<0.001	0.177 (0.065, 0.450)	<0.001

Non-adjusted: none; AdjustI: Adjusted for age, sex, BMI; AdjustII: Adjusted for sex, age, BMI, APACHE II, SOFA, WBC, IL-6, diabetes, hypertension, CKD, COPD, pressor agent, CRRT, transfusion. BMI, body mass index; APACHE II, acute physiology and chronic health evaluation II; SOFA, sequential organ failure assessment; WBC, white blood cell count; IL-6, interleukin-6; CKD, chronic kidney disease; COPD, chronic obstructive pulmonary disease; CRRT, continuous renal replacement therapy.

### Non-linear relationship detection

**Figure 2:**
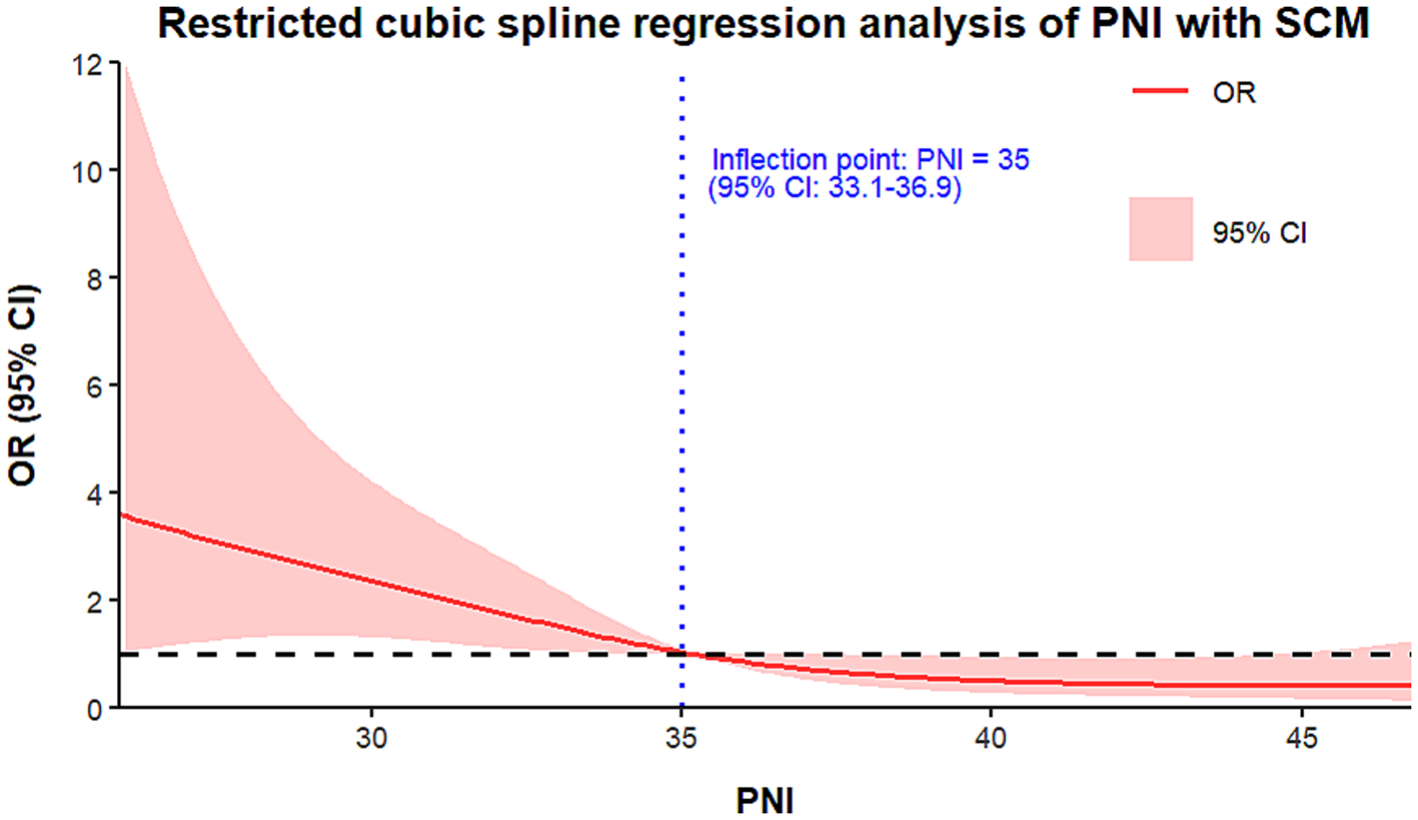
RCS analysis of the association between the PNI and SCM.

The relationship between the PNI and SCM in septic patients was evaluated using RCS regression analysis. The findings indicated an L-shaped non-linear association between PNI and SCM risk. As shown in Figure 2, a clear turning point was observed at a PNI level of 35 (95% CI: 33–37). Each one-unit increase in PNI below 35 was associated with an approximate 10% reduction in SCM risk, with a gradual decrease in OR from nearly 2. Beyond a threshold of approximately 35–40, the OR stabilized near 1, indicating a plateau in the impact of PNI on SCM risk. Logistic regression analysis of PNI tertiles confirmed this association, which persisted after adjusting for clinical factors, including age, sex, ethnicity, heart rate, and respiratory rate. In our cohort of 200 ICU septic patients, a PNI cutoff of 35 yielded a sensitivity of 78.2% and a specificity of 63.5% for predicting SCM occurrence. These findings indicate that the PNI is a key predictor of SCM risk in septic patients, supporting its use for early clinical screening and management.

### Subgroup analysis

**Figure 3:**
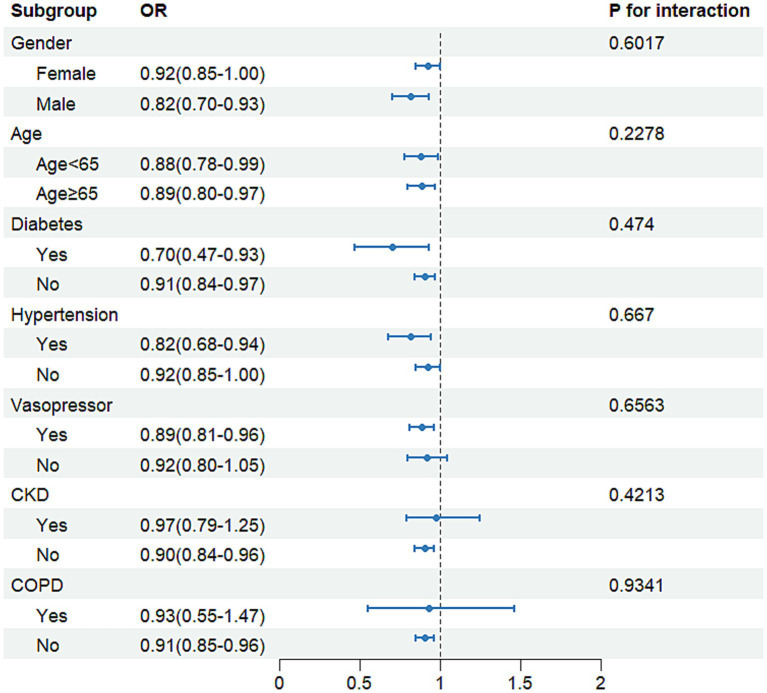
Forest plot of subgroup analysis examining the PNI and new-onset SCM in sepsis.

A subgroup analysis was performed based on hypertension, diabetes mellitus, chronic obstructive pulmonary disease, chronic kidney disease, vasopressor use, age (<65 or ≥65 years), and sex (male patients or female patients). This enabled the evaluation of the association between the PNI and SCM occurrence across different subpopulations of septic patients. A significant association between higher PNI values and reduced SCM risk was observed within subgroups defined by diabetes mellitus, hypertension, age, and sex, as shown in Figure 3. Additionally, significant associations were found among individuals without COPD, chronic kidney disease, and vasopressor use. Interaction tests showed no statistically significant heterogeneity among subgroups (*p* > 0.05). Thus, the association between the PNI and SCM was independent of clinical factors, including COPD, chronic kidney disease, vasopressor use, hypertension, diabetes mellitus, sex, and age. The results consistently demonstrated the relationship between the PNI and SCM across various patient subgroups, suggesting potential implications in diverse clinical settings and populations.

## Discussion

This study used multiple analytical methods, including RCS regression analysis, logistic regression analysis, subgroup analysis, and baseline characteristic assessments, to investigate the relationship between the PNI and SCM in patients with sepsis. This association exhibited an L-shaped pattern. These findings may provide clinical guidance for the assessment of risk and prediction of SCM development.

### Mechanisms underlying the association between the PNI and SCM in septic patients

Infection causes a dysregulated host response, resulting in sepsis, a life-threatening condition characterized by organ dysfunction. This process can impair mitochondrial function, reduce metabolic capacity, and lead to malnutrition (13). Critically ill patients often have pre-existing malnutrition upon ICU admission, a condition that significantly worsens clinical progression and outcomes. Malnutrition, whether pre-existing or sepsis-induced, weakens immune function and reduces the host’s capacity to mount an effective defense against pathogens (16). These factors ultimately cause significant alterations in immune responses, including lymphopenia (20).

During sepsis, metabolic disturbances, immune dysfunction, and endothelial impairment in the heart contribute to arrhythmia and ventricular dysfunction (22). Under septic conditions, cardiomyocytes negatively affect myocardial contractility by shifting their primary energy substrate from free fatty acids to glucose, in a pattern similar to that observed in myocardial hibernation after ischemia (23). As a composite indicator integrating nutritional and immunological status, the PNI combines peripheral blood lymphocyte count and serum albumin levels, precisely reflecting this pathophysiological state (19).

Albumin, a key component of the PNI, affects SCM through multiple mechanisms. First, studies investigating sepsis-induced myocardial injury have shown that mitochondrial dysfunction generates excessive reactive oxygen species (ROS), thereby increasing oxidative stress and subsequently causing cell death and tissue injury (24). Albumin directly neutralizes free radicals using its free sulfhydryl group (Cys 34), thereby reducing cellular damage caused by oxidative stress (25). Second, low albumin levels strongly correlate with elevated levels of inflammatory mediators, such as IL-6, tumor necrosis factor (TNF), and interleukin (IL)-1. These mediators can directly damage cardiomyocytes and cause microcirculatory impairment (26, 27). Third, injury to myocardial endothelial cells and the glycocalyx is associated with pathological effects, including microvascular thrombosis, accelerated inflammation and platelet activation, tissue edema, capillary leakage, hypotension, loss of vascular reactivity, microcirculatory failure, and (multi)organ dysfunction (28, 29). Albumin also plays a vital role in maintaining endothelial glycocalyx integrity and vascular barrier function, particularly in patients with increased capillary permeability (29, 30).

Within the adaptive immune system, lymphocytes play a pivotal role. Lymphocyte apoptosis triggered by sepsis leads to lymphopenia, a primary contributor to sepsis-associated immunosuppression (31). Collectively, these mechanisms explain the relationship between the PNI and SCM. Moreover, the L-shaped relationship between the PNI and SCM indicates the existence of a threshold. When the PNI is below 35, simultaneous reductions in albumin and lymphocyte counts significantly elevate SCM risk even with modest declines. However, when the PNI reaches or exceeds 35, the nutritional–immunological status appears to plateau, diminishing further marginal benefits for myocardial protection and attenuating the PNI’s ability to further reduce SCM risk.

### Epidemiological investigation of the association between the PNI, sepsis, and SCM

Previous studies have demonstrated a significant association between PNI levels and the onset and progression of cardiovascular diseases. Research has indicated an inverse correlation between the PNI and major adverse cardiovascular events (MACE) (32). Furthermore, the PNI is negatively correlated with the risk of heart failure, highlighting its potential as a valuable clinical assessment tool (33). Additionally, a strong relationship exists between PNI levels and atrial fibrillation recurrence in acute myocardial infarction patients undergoing interventional procedures (34). This study identified an L-shaped non-linear relationship between the PNI and SCM incidence in septic patients, suggesting that maintaining optimal PNI levels may preserve cardiovascular function in this population.

### Clinical significance of the study

The PNI, which is derived conveniently from peripheral blood lymphocyte count and serum albumin, exhibits an L-shaped and markedly inverse relationship with SCM risk (threshold ≈35), underscoring its clinical utility. The PNI allows rapid stratification of SCM risk among septic patients. A prioritized screening protocol, including point-of-care echocardiography to measure left ventricular ejection fraction (LVEF) and NT-proBNP testing, is recommended for patients with a PNI of <35, enabling early SCM detection. Targeted interventions, including albumin supplementation, optimized nutritional support, and immune modulation, can be initiated for patients with a low PNI based on their nutritional and immune status to reduce SCM risk. Subgroup analyses confirmed the stability of this association across sex, age, hypertension, and diabetes mellitus subgroups, indicating broad applicability and potential for a unified clinical assessment standard. This approach facilitates efficient ICU resource allocation, prevents overtreatment, and may reduce SCM incidence and overall mortality in septic patients, thereby providing a practical foundation for SCM management.

### Limitations

This study was a single-center retrospective analysis based on electronic medical records, which may contain incomplete data. In particular, variability in laboratory testing times may also have introduced measurement bias. Although missing data were addressed using multivariable adjustment and multiple imputation, unmeasured confounders may still influence the results. Caution should be exercised when generalizing these findings to a broader sepsis population, as the sample size was limited to 200 patients from a single ICU, and complex cases, such as those involving malignant tumors, were excluded.

Furthermore, the reliance of the PNI on serum albumin and lymphocyte count requires cautious interpretation in critically ill patients. Albumin, a negative acute-phase reactant, is significantly affected by systemic inflammation, which may lead to the overestimation of nutritional deficiency (15). These limitations suggest that future studies should explore alternative biomarkers less influenced by acute inflammation and organ dysfunction to improve the clinical utility of the PNI. Therefore, the PNI may not be suitable for patients with primary synthetic or immune dysfunction, such as those with severe hepatic insufficiency or hematological disorders. This limitation formed the basis of the exclusion criteria (15, 19).

Additionally, a PNI assessment based on a single measurement at admission does not reflect the dynamic changes in nutritional and immune status that occur during treatment, which may influence myocardial injury. The diagnostic criteria for SCM, based solely on LVEF and NT-proBNP, may lead to misclassification, as more sensitive indicators such as myocardial strain or injury biomarkers were excluded. Moreover, despite the observed statistical association between the PNI and SCM, mechanistic validation is lacking, and the proposed PNI cutoff and its L-shaped relationship require confirmation in multicenter prospective studies. Notably, the effectiveness of intervention strategies based on PNI remains unclear. Future research should include larger and more representative samples, dynamic monitoring, and mechanistic investigations to enhance the clinical value of the PNI.

## Conclusion

An L-shaped relationship between the PNI and the risk of SCM was identified in this study. In ICU patients with sepsis, the PNI may serve as a useful indicator for predicting SCM development.

## Data Availability

The raw data supporting the conclusions of this article will be made available by the authors, without undue reservation.
